# One Biology and the Status of Humans

**DOI:** 10.3390/ani16010086

**Published:** 2025-12-28

**Authors:** Donald M. Broom

**Affiliations:** Department of Veterinary Medicine and St Catharine’s College, Cambridge University, Madingley Road, Cambridge CB3 0ES, UK; dmb16@cam.ac.uk

**Keywords:** one biology, one health, one welfare, cognitive ability, human status, morality

## Abstract

Biology overlaps for humans and non-humans; so, there is only one biology and humans are animals. All human biological functions can also be found, to some degree, in other animal species. Examples mentioned are DNA and characteristics; cell type and structure; efficiency of muscles and other locomotor mechanisms; efficiency of immune and other body protection mechanisms; sensory functioning; cognition and memory; specific cognitive abilities; and ability to have moral concepts and behave in a moral way. What is the current status of humans? Humans are destroying many other species in the world, as well as whole habitats and ecosystems. Since resources important for humans are amongst those being harmed, and there is, at present, little sign of sufficient change in human behaviour, humans themselves are amongst the species that could be destroyed. Long before this happens, immoral damage to many non-human individuals and much of the rest of the world is occurring. The change in attitude needed for all humans is to place less value on immediate human benefit and more value on benefit for our fellow beings in the world. When the word ‘we’ is used, it should not just include humans. It should, at least, include all sentient beings.

## Simple Summary

Our understanding of the world, our preservation of it, and our species have been harmed by the view that humans are fundamentally different from other animals. A range of areas of animal biology have been considered and it is difficult to find any biological function that is solely human or where there is a sharp distinction between humans and all other animal species. Biology overlaps for humans and non-humans; so, there is only one biology and humans are animals. To emphasize that humans fit closely into the biology of other animals does not contradict the key principles of ethics. Some of the impacts of exploitation by humans have effects on populations of living organisms or on physical qualities of the world environment that have the potential to reduce human resources or even prevent the continuation of human life. There should be a reduction in activities that over-emphasize the importance of humans and an increase in strategies that have a balanced view of humans in relation to all other species in the living world. Another way to state this is that in every human activity there should be moves towards sustainability, taking account of all of the components of sustainability.

## 1. Introduction

Much scientific and other discussion, even by biologists, has emphasized that humans are different from all other species. This has been proposed [1] as part of a more general “defining force in the shaping of human identity: a person’s need to feel special and different from others”. The idea of a ladder or scale of life with humans at the top led to erroneous concepts like animal species being higher or advanced rather than lower or primitive, which is not supported by detailed studies investigating evolution of bodily systems, for example, that of hearing [2]. While every species is unique, is human uniqueness greater than that of other species? A major theme of this paper is that the answer is no. ‘One biology’ means that the concept of biology has exactly the same meaning for humans and for all non-human species of living organisms. Some of the similarities are between humans and plants and bacteria, not just between humans and other animals. However, plants and bacteria are functionally very different from animals because they do not have a nervous system. Almost all biological processes in humans are the same as processes in some other animals. The change in thinking resulting from this biological fact has consequences for human interactions with the rest of the world. While humans may reasonably and biologically feel support for their own species, biased scientific and other writing overstates the importance of humans and this has led to thoughtless exploitation and some of the greatest harms that humans have caused to the world.

Is human biology different from or better than biology of other species? Some say yes and some say no. In arguing that it is, there are authors [3] who describe human success and impressive ability, for example the large biomass of humans and “a spectacular evolutionary anomaly” when the human species appeared. Others [4] concede that “science has taught us that we humans are animals” but, when considering “what makes humans unique” they note that complex abilities “developed much further in our species than in any other”. The early naturalists [5,6] emphasized that “we and the beasts are kin” and explained the high level of intelligence in a range of species. Beliefs held for generations and based upon observations by people in many human societies presented non-human animals as being very similar to humans [7,8] and developments in science have confirmed many of these early observations. Despite this, many have presented reasons why humans are ‘special’. Bingham [9] says that humans “display a highly derived social adaptation involving extensive cooperation among non-close kin. Further, humans possess adaptive capabilities, including language, high cognitive function, and technological virtuosity not previously seen on this planet.” Hill et al. [3] refer to “extensive reliance on social learning, resulting in cumulative adaptive change in extra-somatically stored information” and “extraordinary cooperation between non-kin, including specialization, a regular flow of goods and services between individuals and groups, and the formation of increasingly complex alliance networks.”

The processing capacity of the human frontal and pre-frontal cortex is greater than that of other animals. Mathematical logic, perception of time, complex reasoning, analytical capacity, and prediction of events are better in humans than in most other species. However, these are differences in degree, not in absolute capability [10]. This paper reviews a range of biological studies and then considers the consequences of the reported bias and what might be done about it. The focus is on producing a balanced view and some of the very large number of publications that present the view that humans are very different from all other species are cited. The balance that is sought is not in relation to numbers of publications but a scientific balance. The concept that there is only one biology [11,12] is, in my view, a consequence of the best quality science, while the idea that humans are extremely different from all other animals and have different biology is based on illogical prejudice and on misunderstanding, incorrect evaluation, or lack of consideration of scientific evidence.

## 2. Aspects of Biology Where Humans Might Be ‘Special’

**Figure 1:**
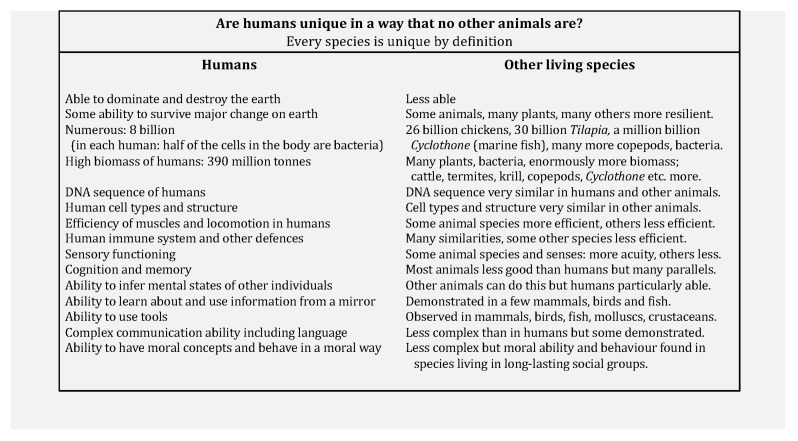
Summary of abilities where humans are said to be unique to a degree greater than for all other species.

A summary of some of the ways in which humans have been presented as unique is shown in Figure 1 and more details of some of the biological evidence [13] are reviewed below. An issue that is difficult in a discussion of whether or not one example or species is different from a set of examples or species is how great should the difference be in order to consider it a category changer? At what point would it be logical to say that there is more than one biology? The scientific position presented here is that there is not enough difference to justify saying that human biology is different from the biology of all other species.

### 2.1. DNA and All Characteristics

Much biological research, whose aim is to find out about the functioning of humans and other species, has concentrated on animals considered convenient for laboratory use, especially the zebrafish *Danio rerio*, the mouse *Mus musculus*, the brown rat *Rattus norvegicus*, the nematode worm *Caenorhabditis elegans*, and the fruit fly *Drosophila melanogaster*. In nerve cell function, the long-finned squid *Loligo forbesii*, and in analgesic and anaesthetic research, the marine mollusc *Aplysia* have been much used. These animals have been used for laboratory convenience. However, the idea underlying their use to find out about fundamental biological functioning and human functioning is that there is much similarity in mechanism across a wide range of animal groups [14].

As the DNA of more and more species is sequenced and described, it has become clear that the genetic differences between humans and other species of animals are small and the similarities are large [15]. The same methods of DNA analysis are used for humans and non-humans, for example, in forensic work [16]. Mitochondrial DNA sequences generally evolve more rapidly than those of nuclear genes. Complete mitochondrial genome sequences provide a set of “genome-level characters” and the relative arrangements of genes can indicate common ancestry [17]. However, the overriding results of analyses indicate similarities across species [18]. The significance of the small difference between the human genome and that of other species has been discussed [19], for example, in relation to small-scale and large-scale segmental deletions and duplications and the complexity of translating genotype to phenotype. The high degree of similarity between human and other genomes remains a difficulty for those proposing that humans are unique in a way that other species are not.

The way in which genetic information is utilized by organisms is very similar in humans and all other species. It is now known, especially from epigenetics and other research, that while all characteristics of organisms depend on genetic information, each step in the translation of genetic information into proteins, messenger RNA production, etc., and hence, characteristics of individuals can be altered by environmental factors [20,21]. If the terms “genetically determined,” “instinctive,” and “innate” mean independent of environmental factors, nothing is completely genetically determined, nothing is instinctive, and nothing is innate. Humans are animals and have hardly any ability that is not also possessed by other animal species, at least to some degree.

The study of evolutionary divergence is relevant to understanding how much humans diverge from other mammals or mammals diverge from other vertebrates and invertebrates. Striedter [14] cites the concept that the mean difference between species increases with the square root of evolutionary divergence time, rather than linearly [22], and it also explains factors affecting vertebrate protein divergence rates. For example, genetic lineages with short generation times tend to diverge faster than others. It is desirable for biologists to conduct their research on a range of different species, and studies of what causes divergence are of value. However, a general conclusion is that functional similarities across taxonomic groups remain impressively high. It is thought by some that the enormous diversity among species in the total amount of DNA is an indicator of organismic complexity, but this is clearly not correct [23,24]. Some of the DNA is of core importance in relation to function and varies little across species.

### 2.2. Cell Type and Structure

There are many cell types, identifiable by structure and function, which occur in a wide range of animals, including humans, and each type may be considered to have a core regulatory complex [21]. In a review of a wide range of cell types in mammals [25], there is scarcely any difference between human and other animal cells. There are analytical methodologies that can be utilized in studies of a wide range of species; for example, for central nervous system cell types, the use of translating ribosome affinity purification is suitable for humans, all vertebrates, and all invertebrates [26,27]. Microglial cells in the brain vary little in density and in basic structure across a wide range of species [28]. Among muscle cells, although there are differences between some vertebrates and some invertebrates, there are also great similarities, for example, the smooth myocytes in the bilaterian visceral muscles in vertebrates and in annelid worms [29,30].

### 2.3. Efficiency of Muscles and Other Locomotor Mechanisms

In studies of the efficiency of striated muscles when producing positive work in a range of vertebrate species, it was found that efficiency increases with speed and that the peak efficiency increases with body size [31]. The animal classificatory group had little effect. When contraction was examined in detail, there were differences in that the highest initial mechanical efficiencies were in muscles of tortoises and mussels, while mammalian muscles appeared to be less efficient than the muscles of frogs, toads, and tortoises. However, the greater efficiencies seemed to be a consequence of requirements for survival during life rather than taxonomic group differences. Humans and other mammals were not superior [32]. Most of the variation in heart design, cellular bioenergetics, and mitochondrial efficiencies in humans and other mammals are associated with body size and the environment lived in rather than the function being better in humans [33], while heart disorders and the drugs used to treat them are mostly the same in different mammals, for example, in cats and humans [34,35].

Comparison of the energy cost of pedestrian locomotion shows substantial differences with size and with the extent of other adaptations, for example, those for swimming, but not systematic variation across animal groups [36]. While humans may be quite well adapted for long-distance running [37], their efficiency in movement is not unusual and animals that can fly or swim well have great advantages over humans. Humans are said to be adapted for running in that they have many sweat glands, but several factors seem to affect sweat gland distribution [38,39].

### 2.4. Efficiency of Immune and Other Body Protection Mechanisms

Mechanisms whose function is to prevent damage to the organism by foreign substances and pathogens exist in all animals, and the immune system has substantial uniformity across all vertebrates [40]. Immune defences are commonly divided into innate and adaptive, where innate does not mean independent of all environmental effects but refers to systems that do not require prior exposure to a specific pathogen or antigen in order to function. Innate immunity, the first line of defense characterized by pattern recognition receptors and immediate responses, is present across the entire animal kingdom from invertebrates to mammals [41]. Sponges and cnidarians possess toll-like receptors and antimicrobial peptides that function similarly to those in humans [42]. The mechanisms of phagocytosis, inflammatory response, and complement activation show remarkable conservation across diverse taxa, suggesting ancient evolutionary origins for these core defensive strategies [43].

Adaptive immunity, characterized by immunological memory and specificity, shows a more complex evolutionary trajectory but still defies the notion of human exceptionalism. All jawed vertebrates possess an adaptive immune system characterized by diverse antigen recognition receptors. Agnathan chordates, which preceded fish in appearance on earth, have variable lymphocyte receptors that show functional similarities with the essential features of lymphocyte-based adaptive immunity shared by jawed vertebrates, despite using different molecular mechanisms [44]. The recombination-activating genes that facilitate the enormous diversity of antibodies in mammals evolved from transposable elements present in early deuterostomes [45]. The remarkable similarity of the immune systems of vertebrate species has resulted in the widespread use of rodents, such as mice and rats, as a mainstay in biomedical research and pre-clinical testing of immunomodulatory agents, where it is useful to study effects on an intact immune system [46].

In both vertebrates and invertebrates there is functional interaction between the brain, endocrine, gut, and immune systems [47,48,49], with evolutionary conserved pathways through which neuroendocrine signals modulate immune responses across diverse species while certain animals possess specialized immune adaptations suited to their ecological niches. For example, some deep-sea fish have evolved distinctive antibody structures with exceptional conformational flexibility. These differences represent variations in degree rather than fundamental distinctions in organization and function.

### 2.5. Sensory Functioning

Humans are particularly aware of information from vision but many species have greater visual acuity, which depends mainly on body size and nocturnal or diurnal living [50]. Time perception of flicker during vision or of other detectable sensory fluctuation is affected by body size and metabolic rate; so, human ability is largely predictable from these variables rather than being a special human characteristic, and small animals have better ability than large animals [51].

Mammalian hearing is also influenced by body size. High-frequency hearing is proportionally related to body size; so, humans, being large, have very restricted high-frequency hearing compared with most other mammals but relatively good sound localization abilities [52]. Human hearing selectivity is higher at lower frequencies; so, the sounds utilized during speech may have evolved to be at these frequencies for that reason [2]. Many non-human animals can hear higher or lower frequency sounds than humans can [53]. Echolocation is of great importance in several non-human species and some deaf humans can use it [54]. The sense of taste in humans seems to be utilized in a similar way to its use in other animal species [55].

Olfaction is less sensitive and narrower in discriminatory range in humans than in many domestic and wild mammals, fish, and insects [56]. However, recent work shows that humans use olfaction more than had previously been thought [57]. Detection of water pressure, magnetic field, and electrical field is sophisticated in fish and other aquatic animals but minimal in humans [58].

### 2.6. Brain, Cognition and Memory

Very many people, mostly non-scientists, have assumed that brain size determines cognitive ability and, partly because brain size increased during human evolution, that humans have the largest brains. Humans do not have the largest brains of all animals, nor the largest brain in relation to body size, and the complexity of brain function is not related in a simple way to brain size. Also, the region of the brain where complex analysis occurs varies across animal species; so, a large cerebral cortex is not essential for high level processing. As brain function has become a topic investigated experimentally [59], the similarities between humans and non-humans have become more and more apparent. Much brain function is involved in interpreting sensory information, including, for example, the parietal cortex, which draws together information from somatosensory, visual, and other inputs in mammals and other vertebrates [60]. Macphail [61] argues that there are quantitative but not qualitative differences between cognitive abilities in vertebrate species. For example, Lind et al. [62] reviewed studies of delayed matching-to-sample studies in a range of mammals, birds, and bees. They concluded that there was no evidence for differences in this respect, or in the general area of duration of memory. In particular, monkeys and apes were not better than other animals. Hahn and Rose [63] review studies of working memory and reported that it is comparable across vastly different species and that specific adaptations are a consequence of their respective ecological niches and local contextual variables. While ‘neuroecology’ may not wholly explain any aspect of brain function, diversity in brain function can usefully be discussed in relation to such factors [64]. Studies of cognition and memory in one species can often provide useful information about another, for example, in sheep and humans [65]. As Emery [66] points out, elements of a wide range of cognitive abilities can be found in many species and the evidence acquired via sophisticated phylogenetic techniques can be used to reconstruct the evolution of cognition.

Human cognitive abilities are impressive, but more and more recent research shows that the differences between humans and other species are in degree rather than in categorical ability. Mirror neurons, which have been described in the brains of humans, other primates, and some birds, allow sophisticated social interactions. Some people think that having a cognitive representation of an object or other resource independent of current sensory input is a uniquely human characteristic. For example, at one time, it was thought that a chicken would lose any concept of an object if it were out of sight. However, not only can young domestic chicks go to objects hidden behind screens, but when two or three objects were hidden behind screens, the chicks went to the screen with the larger number of objects [67]. A visual or auditory symbol can be used by many species. Langbein et al. [68] were able to train goats to obtain water by carrying out a specific action when they saw one particular picture rather than others. In another carefully controlled study, a dog was trained to point to or fetch one of several objects, such as a ball, a stick, a bottle, a key, or a toy bear, when she heard a human word relevant to that object. This dog also indicated using symbols on a keyboard that she wanted ‘water’, ‘food’, ‘stroke me’, ‘I go out’, ‘I get a toy’, or ‘I urinate’ [69]. There are reviews of cognition in domestic and other animals, including abilities to predict human actions [8,70,71].

### 2.7. Specific Cognitive Abilities

Language, high cognitive function, and technological virtuosity are said by some to be uniquely human [9]. Some discussions comparing awareness in various species consider whether or not the members of a species have the cognitive ability that humans have to infer the mental states of others [72,73]. The term ‘theory of mind’ may be used, but the distinction between mind and brain is not useful [7]. Two examples of relevant studies on non-human animals include firstly, pigs observing another pig finding food and later copying what that pig did to get food [70] but not doing so if a third pig that might have robbed it was watching [74]; and secondly, dogs that had seen a toy hidden in an area that they could not reach subsequently signaling where the toy was to a human helper who obtained it for the dog [75]. Such ability to deduce what others know and to use this information increases the likelihood of showing empathy [76].

After a certain stage of development, humans and several non-human species show the ability to use complex modifications of potential sensory input, for example, a mirror. Pigs given five hours of experience with a mirror used the image in a mirror to find concealed food whose location was evident only from the mirror [77]. Tests with chimpanzees, an elephant, dolphins, and magpies with previous experience of mirrors, using marks on the body visible in a mirror, led to the individuals touching or apparently looking at the marks [78,79,80,81,82]. Cleaner fish *Labroides dimidiatus* given experience of a mirror were able to use it to distinguish themselves from images of other fish [83], to evaluate their own size, and to respond accordingly to photographs of conspecifics [84].

At one time, it was thought that only humans select, design and use tools. However, Reader and Laland [85] found 607 reports of tool use in primates. Over sixty years ago, sea otters were described obtaining stones from the sea floor and bringing them to the surface to break shells and obtain the food within [86]. Individual sea otters that use tools in this way have been shown to have greater foraging success and better tooth health [87]. Chimpanzees and crows have been reported to use a tool to reach a second tool that could be used for accessing food [88,89], while several aquatic species have been observed to collect and use objects [90,91]. Analysis of studies of tool use in primates and corvids shows more diversity in tool use reported for primates, but this may be because a greater variety of studies has been carried out on primates [92]. Humans show complex and very diverse tool use, sometimes using tools in sequence, but a careful comparison of chimpanzee and human hunter gatherer tool use concluded that while humans used tools to make tools and used fire, there were few differences in tool use to obtain food [93]. Young chimpanzees pay close attention to conspecifics at the stage when they learn to use tools, and groups of chimpanzees remember and revisit army ant nests over many years [94,95]. Many actions during the development of humans and other animals include components of tool use and have relevant neural processes [96].

Humans are particularly able in their capacity to discriminate ordered sequences of stimuli [97]. This ability is relevant to capability in language and to aspects of social organization. Even children of a few months of age have been shown to have such ability [98] but, to some degree, other species also have it. Mimicry requires sophisticated sequential discrimination and appreciation of context. It has been described in many non-human species, and it can lead to local dialects in production of sounds and other communication possibilities. Another human response that communicates to other individuals is laughter, but very similar responses to similar stimuli have been described in rodents, cetaceans, and other primates [99]. Again, the understanding of sequences is complex. The idea that there was a dramatic transformation from non-human communication to human language, associated with a major increase in complexity of cognitive function, is controversial and has few scientific supporters [100]. Tattersall [101], considering the origins of human language, points out that the evidence for symbolic thought in humans appears after the evolutionary advent of the identifiable species *Homo sapiens*, by which time, the anatomy needed to produce words was already in place. Efforts to consider parallels with communication in other species are clearly needed.

It has been suggested [2] that chimpanzees lack communication ability of human quality, because those raised in human families learn a few hundred words but no grammar and they do not share food. However, there are other possible developmental reasons for these results [102]. Chimpanzees and orang-utans do modify their visual and auditory signals to humans according to the current situation and the reward context [103]. Many other social mammals and birds have complex visual signaling systems that are comparable to aspects of human language. While language can be defined in human-centered ways [104], other biologically focused definitions do not indicate human uniqueness. Much human communication is non-verbal and similar in complexity to some verbal communication and the same is true of complex non-human communication. Several primate species can respond to human words, or combinations of human words, and parrots can use combinations of human words to obtain resources or describe situations. The use of some degree of syntax and grammar in complex communication situations by non-human species would seem to overlap substantially with human language. Barón-Birchenall [105], in a comprehensive review, argues that there is a certain degree of continuity between different aspects of human language and non-human animal communication systems, in different domains including the syntactic. Human language is impressive and sophisticated, but communication in some other species is also very complex in different ways.

Language is an aspect of culture which involves the transmission of ideas and methods within societies. Animals of a very large number of species learn from others and some develop culture over generations, but humans do this to a greater extent than other species [106]. Culture is certainly not unique to humans, although human culture may be able to change faster than in other species [107]. In this area, as with all others described here, there are differences in degree between human and non-human abilities, but aspects of the human ability are evident in other species.

### 2.8. Ability to Have Moral Concepts and Behave in a Moral Way

While it has been suggested that only humans cooperate and form coalitions with non-kin [9], some degree of cooperation among individuals that are not close kin, sometimes lasting for a substantial proportion of life, has been described in thousands of scientific publications concerning social mammals, birds, and fish, and some papers describe coalitions. In order that a long-lasting social group can be stable, there must be cognitive function, behaviour, and other mechanisms that minimize or prevent actions that tend to destabilize the group [7,108,109,110,111]. The consequent set of brain concepts and abilities can logically be called morals.

Humans are not the only animals that behave in a moral way, as shown by many years of study of a wide range of social animals. Some of the mechanisms that make moral behaviour possible are promoted by and form the basis for the development of the major religions [7,111,112].

Some non-human animals are thought of by many people as moral subjects and by some as moral agents [113,114]. Amongst the abilities required that promote moral behaviour are altruism and empathy. While altruism is widespread across species, reciprocal altruism is detectable only by use of long-term investigations. Many of these investigations have been carried out and altruism has been described in several species [115]. Empathy has a biological basis which exists in many non-human animals [7,116]. Empathy alters functioning and motivational state [117] and compassion is more likely to be shown to those perceived to be in need of empathy [118].

## 3. One Health, One Welfare, One Biology

The terms health and welfare apply to all animals, and the One Health concept makes it clear that health means exactly the same for non-human animals as it does for humans. The worldwide One Health strategy [119,120] encourages interdisciplinary collaboration and communication in all aspects of health care for human and non-human animals. It advocates that the individual human, or other animal, should be viewed in relation to all of its interactions with the environment. This approach encourages thinking about non-human animals as individuals and hence having some intrinsic value in the world.

The welfare of an individual is its state as regards its attempts to cope with its environment [12,21,121]. Just as with One Health, the One Welfare approach emphasizes that the concept of welfare is identical whether applied to humans or to non-human animals [122,123,124,125,126]. This approach is being incorporated into the teaching of animal welfare [127]. Progress in understanding the welfare of humans and other species is slower if research on humans is thought to be quite different from that on other animals. A further message from One Welfare thinking is that when the welfare of individual humans or non-human animals is poor, there is increased susceptibility to disease; so, improving welfare generally reduces disease. Since there are many diseases and pathological conditions that affect both humans and other species, those with a medical background and those with a veterinary or other biological background benefit from exchanging information, as was advocated over twenty years ago in a Dahlem conference [128]. There are many areas where understanding pathologies across species can be thus facilitated; for example, Daigle [129] described similarities between post-partum problems in pigs and humans. Individual rather than herd treatment may be needed to provide good care for people and good care for animals used by people. A further key idea for all species, since health is the state of the individual in relation to coping with pathology, is that health is an important part of welfare, not a separate topic [12,21].

As explained, health means the same for humans and all other animals, and welfare does as well. The reason for this is that a high proportion of biological processes are the same across very many species and there is only one biology. Health and welfare are biological concepts and the strong arguments for their meanings being the same for human and non-human animals are grounds for revisiting the whole concept of biology. All aspects of biology should be used in the same way for all animals, including humans. Section 2 of this paper details various arguments for human exceptionalism. While, as stated in Figure 1, every species is unique by definition, the biology of humans overlaps very greatly with the biology of other species.

If humans and other animals are similar, is there anything about human life and activities that should be changed? It would seem that it is scientifically unsound to treat humans as if they were completely different from all other species. One consequence is for language. The use of biological terms by all people should be more precise [130]. Secondly, when forming strategies for how to live in the world, every person should reduce activities that over-emphasize the importance of humans and endeavour to have a balanced view of humans in relation to all other species in the living world. For many generations, most humans have believed that the living world exists for human benefit and have often exploited aspects of it without regard for consequences such as causing poor welfare in other species or drastic reduction in populations of other species, even to the point of extinction. Arguing for similarity between humans and other species does not mean that humans should not use other species but does mean that human impacts on other species should always be considered. There is widespread public demand that the many components of the sustainability of systems for production and other human actions, including the welfare of animals used in or affected by the production, should always be taken into account [131,132] and that food and other products should have labels that indicate this [133]. This is evidence for changing morals as biological knowledge becomes more widely known. Each person can consider whether or not it is justifiable to cause loss of life or poor welfare of non-human animals when driving a vehicle, spreading herbicide or any other chemical, interacting with animals considered to be pests, purchasing a product, or engaging in some form of entertainment or any other activity. Some of the impacts of human exploitation have effects on populations of living organisms or on physical qualities of the world environment that have the potential to reduce human resources or even prevent the continuation of human life [134,135,136]. A fundamental aspect of this discussion is the question of the intrinsic worth of each individual, human or non-human. The arguments put forward in discussions of one health and one welfare can readily be related to intrinsic worth [137].

## 4. Conclusions

A range of biological characteristics of animals are considered in order to compare humans and other species. In relation to every characteristic, there are some differences across species. However, in the efficiency of cellular and immune system functioning and cognitive abilities such as use of a mirror, use of tools, and language, there are either clear examples of the same ability in some non-human species as in humans or a gradation of ability. It is difficult to find any biological function that is solely human or where there is a sharp distinction between humans and all other animal species. Biology overlaps for humans and non-humans; so, there is only one biology. What is the current status of humans? Humans are destroying many other species in the world. Humans are destroying whole habitats and ecosystems. What will be the consequences? Since resources important for humans are amongst those being harmed, and there is, at present, little sign of sufficient change in human behaviour, humans themselves are amongst the species that could be destroyed. Long before this happens, immoral damage to many non-human individuals and much of the rest of the world is occurring. The change in attitude needed for all humans is to place less value on immediate human benefit and more value on benefit for our fellow beings in the world. When the word ‘we’ is used, it should not include only humans. It should, at least, include all sentient beings.

## Data Availability

No new data were created or analysed in this study.
